# A Numerical Study of the Dynamics of Vector-Born Viral Plant Disorders Using a Hybrid Artificial Neural Network Approach

**DOI:** 10.3390/e24111511

**Published:** 2022-10-22

**Authors:** Hosam Alhakami, Muhammad Umar, Muhammad Sulaiman, Wajdi Alhakami, Abdullah Baz

**Affiliations:** 1 Department of Computer Science, College of Computer and Information Systems, Umm Al-Qura University, Makkah 21955, Saudi Arabia; 2 Department of Mathematics, Abdul Wali Khan University, Mardan 23200, Pakistan; 3 Department of Information Technology, College of Computers and Information Technology, Taif University, Taif 21944, Saudi Arabia; 4 Department of Computer Engineering, College of Computer and Information Systems, Umm Al-Qura University, Makkah 21955, Saudi Arabia

**Keywords:** mathematical modeling, artificial neural networks, numerical solutions, delay differential equations, optimization techniques, machine learning, Levenberg—Marquardt algorithm

## Abstract

Most plant viral infections are vector-borne. There is a latent period of disease inside the vector after obtaining the virus from the infected plant. Thus, after interacting with an infected vector, the plant demonstrates an incubation time before becoming diseased. This paper analyzes a mathematical model for persistent vector-borne viral plant disease dynamics. The backpropagated neural network based on the Levenberg—Marquardt algorithm (NN-BLMA) is used to study approximate solutions for fluctuations in natural plant mortality and vector mortality rates. A state-of-the-art numerical technique is utilized to generate reference data for obtaining surrogate solutions for multiple cases through NN-BLMA. Curve fitting, regression analysis, error histograms, and convergence analysis are used to assess accuracy of the calculated solutions. It is evident from our simulations that NN-BLMA is accurate and reliable.

## 1. Introduction

Plant disease epidemiology studies how diseases affect plant populations and how to combat plant diseases. Using spatial and temporal plant epidemiology models can provide useful statistical and mathematical data about disease transmission. In the mid-20th century, plant epidemiological models became prominent [1]. Examples of actual uses of this type of model include cassava mosaic disease [2], pine wilt disease [3], and potato late blight [4]. Later, new methods for studying nonlinear dynamics and numerical simulations helped solve complex ecological problems [5,6]. This accelerated the creation of more realistic and complicated plant disease models.

An essential part of the plant epidemiological system is modeling the interactions between infected and healthy plant populations, either directly or via a vector. Infected vectors feed on healthy plants, infecting them. Similarly, non-infected vectors become infected by diseased plants. The vector-borne plant disease is classified as persistent, semipersistent, or non-persistent based on the infectious agent’s residence period in the vector [7,8]. The vector ingests viruses while feeding on infected plant sap in persistent transmission. The salivary glands then release the viruses into the plant tissue as they penetrate the digestive system. The persistent mode of transmission differs from the other two because it takes a long time for a vector to become infected with the virus and become infectious [7,9]. In the case of vectors, this time lag is referred to as the latent phase of infection.

The latent period in plants is similar to the time it takes for a healthy plant to become infected following infection [10]. The incubation period (or incubation time) is the time it takes for symptoms to manifest following infection [1]. Depending on the plant species, the incubation period varies [11]. Incubation durations for beet mosaic virus (BMV), African cassava mosaic virus (ACMV), tobacco mosaic virus (TMV) and bean golden mosaic virus (BGMV) are 7–15 days [12], 3–5 weeks [13], 5 h [14], and 5–6 days [15], respectively. The incubation and latent periods in plants are distinct. However, the expression of disease symptoms correlates with disease transmission [16]. Furthermore, determining the latent period is challenging, whereas observing disease signs is straightforward. So our model development analysis considers the incubation period.

Among the most frequent vector-borne viral diseases affecting crops, leaf curl disease and mosaic disease are two of the most common. The whitefly (*Bemisia* sp.), which transmits several viral infections to Jatropha, cassava, tomato, tobacco, cotton, and other plants, is a hemipteran vector. Most of the disease is systematically spread by whiteflies, meaning that a latent period is frequently observed [17]. Unfortunately, information on the latent and incubation time of infection for various persistently transmitted diseases is lacking in the literature. Due to the variety of viral agents and host plant species, both delay methods have varying effects on disease severity. It also differs between whitefly species and host plants. These delays may vary due to genetic complexity, climate fluctuation, phenotypic heterogeneity, and plasticity [18]. The plant incubation period is usually longer than the latent period in vectors. For example, ACMV has a 6-hour latent period and a 3–5 week incubation period [13].

Ordinary differential equations (ODEs) models cannot account for the incubation or latent period. However, models based on delay differential equations (DDEs) allow system integration. It can represent a system’s dynamics when its evolution depends on prior events. When time lag responses exist, delays are one of the most powerful mathematical modeling tools [19]. DDE models are more sophisticated than ODE models but more realistic. Prey–predator mathematical models with delay differential equations are commonly employed [20,21]. Delay can teach us dynamic phenomena, such as instability, oscillations, and bifurcation.

Van der Plank [1] used DDE to delay plant epidemics. Cooke [22] proposed a model with an incubation time state variable for vector-borne diseases. Wang et al. [23] discussed wheat starch and gluten’s thermal characteristics and interactions. Zhang [24] added the plant incubation period to a Meng and Li [25] plant disease model, causing modifications in the model’s dynamics. Munyasya et al. [26] proposed an integrated on-site and off-site rainwater-harvesting system that enhances rainfed maize output for better climate change adaption. Buonomo and Cerasuolo [27] presented and analyzed a soil-borne plant disease dynamics model. Miao [28] suggested an accuracy of space-for-time substitution for predicting vegetation status after shrub restoration.

An ODE model of the impact of replanting and roguing on eliminating plant disease latency comprises a compartment for latently diseased plant populations [29]. The model does not consider any vector compartment, but it includes classes of latently infected, healthy, post-infection, and infectious plants. Holt et al. [2] proposed a model with infected plants, healthy vectors, and susceptible vectors but no delays. The vector-borne plant disease model [30] was modified by Jackson and Chen [31] by delaying plant incubation and vector latent periods. The threshold value for delay-induced destabilization was determined by observing changes in system solution dynamics. Li et al. used an updated model [31] to analyze Hopf bifurcation, which included incubation and latent period characteristics [32].

Banerjee and Takeuchi [33] identified several critical elements of the dynamics that could lead to false findings. A long wait can stabilize or cure a system Buonomo, and Cerasuolo [27]. Transcritical bifurcations, periodic oscillations, and stability switches can be revealed if the vector-borne plant disease models’ parameters change [2,27,34]. The undelayed model analysis cannot be ignored [31,32]. A mathematical model (1) with parameters given in Table 1 [2,35], which was previously analyzed by Basir et al. [35] for persistent vector-borne viral plant disease dynamics for the effect of both latent period and incubation delay of the dynamics of the deceased. This model is numerically analyzed using a gradient-based numerical technique. Numerous studies claimed that gradient-based techniques, such as RK-4, take up much more computer time than soft computing methods with comparable accuracy and that it is difficult to produce accurate global estimates of the truncation error [36,37]. For instance, at each step of the RK-4 method, the derivative must be evaluated *n* times. Here, ’*n*’ is the order of accuracy of the RK-4 method, which is a significant drawback of gradient-based algorithms [38]. Moreover, RK-4 suffers from divergence for complex systems [39]. Failure in the case of singularity is another hurdle in using these gradient-based numerical techniques. Keeping these disadvantages in mind, the authors of this paper aimed to suggest an alternative gradient-free approach that can handle problems, such as model (1), with accuracy and reliability. The key features of this study are outlined as follows:

- In this paper, we analyzed an established mathematical model (1) for persistent vector-borne viral plant disease dynamics, which is presented in Section 2. The set of parameters substituted in the model is for the case of cassava mosaic disease.
- A gradient-free intelligent design of a two-layer artificial neural network architecture and the Levenberg—Marquardt algorithm is utilized to formulate surrogate solutions. A state-of-the-art numerical method is used to calculate reference solutions for establishing the accuracy, validity, and reliability of NN-BLMA; see Section 3.
- The impact of variations in parameters, such as plant mortality and vector mortality rate, on the model of persistent vector-borne viral plant disease dynamics is observed through the surrogate solutions formulated by the designed NN-BLMA; see Section 4. Graphical analysis for the convergence of NN-BLMA is carried out based on mean square error, regression analysis plots, and error histograms. Moreover, statistical values are tabulated to show the accuracy and reliability of the designed technique.

**Table 1:** Parameters’ description and their numerical values.

Parameters	Description	Values	Unit
*r*	Net growth rate of plants	0.3	${\mathrm{time}}^{-1}$
*K*	Carrying capacity	1	$m^{-2}$
λ	Infected vector to healthy plant disease transmission rate	0.025	${\mathrm{vector}}^{-1}{\mathrm{time}}^{-1}$
μ	Plants natural mortality rate	0.1	${\mathrm{time}}^{-1}$
$m_{1}$	Mortality of infected plants	0.01	${\mathrm{time}}^{-1}$
Π	Vector population’s overall growth rate due to immigration or births	40	${\mathrm{time}}^{-1}$
β	Transmission rate between diseased vector and healthy plant	0.03	${\mathrm{plant}}^{-1}{\mathrm{time}}^{-1}$
*d*	Vector mortality rate	0.1	${\mathrm{time}}^{-1}$

## 2. Problem Formulation

This section develops a mathematical model for persistent vector-borne viral plant disease dynamics. The model considers plant and vector populations without explicitly including the mosaic virus. H(t) signifies healthy plants, while the infected plants are represented by I(t), Q(t) represents uninfected, and W(t) represents the infected whiteflies population.

Due to restricted plantation space and natural resources, logistic growth *r* and carrying capacity *K* are considered for healthy plants [2]. A healthy plant becomes infected when it comes into contact with an infected vector. When an infected vector and a susceptible plant are present, λ is the transmission rate, and λHW is the number of sensitive individuals moving from the susceptible compartment to the infected compartment.

An insect pest, such as a whitefly, shifts its host in response to changing biological and environmental conditions. They generally move between fields of crops [40,41]. They breed in the fields. The Holling type III survival curve describes their life course because of the high death rate they experience early on [41]. Whiteflies (adults and nymphs) can transmit illness.

Crops are typically planted and reaped at specific times of the year. Most crops are reaped a few months after they are produced. A few vectors travel from close or distant patches and reproduce in the vegetation. Vectors grow by migrating from another patch because of reproducing in the same patch or vegetative area. For the same reason, seasonal fluctuations in vector populations are ignored [35].

An open system is considered in this model. Assume Π is the rate of vector birth and migration into the system. No vertical virus transmission is allowed, and a vector cannot infect another vector. Viruses do not destroy or defend vectors. The vector retains the virus and does not recover. However, the infective insects do not get sick from the virus [31]. Let the mortality rate of plants and vectors be represented by μ and *d*, respectively. Infection-related plant death is expected to be higher than average plant mortality. $m_{1}$ is the infection-related mortality of infected plants. Thus, the overall plant mortality rate is $m=\mu +m_{1}$. Consider β to be the conversion between uninfected vectors (i.e., *Q*) and the infected plant (i.e., *I*). So, βQI signifies entering the number of uninfected vectors *Q* into the infected vectors *W* compartment.

In truth, both plant and vector infection takes time. Let $\tau_{1}\in R^{+}$ be the healthy plant’s incubation time following successful infection. At time *t*, the disease transmission is given by the expression $\lambda e^{-m\tau_{1}}H\left(t-\tau_{1}\right)W\left(t-\tau_{1}\right)$, where the positive constants described previously are λ and μ. The term $e^{-m\tau_{1}}$ denotes the chance of a healthy plant surviving through the incubation time $\left[t-\tau_{1},t\right]$, i.e., the number of susceptible plants that came into touch with an infected vector at time $t-\tau_{1}$ and lived up to time *t* to become infected plants.

Again the latent period in a vector is $\tau_{2}\in R^{+}$. At time *t*, the expression $\beta e^{-d\tau_{2}}Q\left(t-\tau_{2}\right)I\left(t-\tau_{2}\right)$ describes the transmission of infection, where $e^{-d\tau_{2}}$ reflects the vector’s survival probability across the latent time $\left[t-\tau_{2},t\right]$. The number of uninfected vectors met an infected vector at time $t-\tau_{2}$ and survived until time *t* to become infected [35]. Based on the given assumptions, the mathematical model is

(1) $$\begin{array}{cc} \frac{dH}{\;dt} & =rH\left[1-\frac{H+I}{K}\right]-\lambda HW,\\ \frac{dI}{\;dt} & =\lambda e^{-m\tau_{1}}H\left(t-\tau_{1}\right)W\left(t-\tau_{1}\right)-mI,\\ \frac{dQ}{\;dt} & =\Pi -\beta QI-dQ,\\ \frac{dW}{\;dt} & =\beta e^{-d\tau_{2}}Q\left(t-\tau_{2}\right)I\left(t-\tau_{2}\right)-dW, \end{array}$$

The initial biological conditions are

H(t)>0, I(t)>0, Q(t)>0, W(t)>0; t∈[−τ,0], $\tau =max[\tau_{1},\tau_{2}]$,

The parameters used in the model (1) assigned some numerical values for solving the model numerically, and Table 1 shows its description and numerical values.

## 3. Design Methodology

This section examines artificial neural networks (ANN) using a novel approach to machine learning by focusing on the supervised neuronal learning mechanisms of these networks to utilize the study of the model for persistent vector-borne viral plant disease dynamics.

### 3.1. Artificial Neural Network (ANN)

An artificial neural network is a network of interconnected core components known as neurons that receives various inputs and generates only one output; each neuron represents a mapping. A neuron’s output is a function of the total of its inputs produced by the activation function.

### 3.2. Activation Function

To introduce nonlinear properties, an activation function is used in an ANN. In a neural network, (Xi,Wi) stands for inputs, weights, and f(Xi), which is the input function that is sent to the network’s output. This output function can then be used as an input for any additional layers or the final output [42,43,44].

The number of hidden units can be optimized using a multilayer perceptron (MLP). Both the weights and biases of the connections were enhanced as well. The construction of a standard MLP with one hidden layer is as follows:

(2) $$H_{j}=\sum_{i=1}^{n}W_{ij}X_{i}+b_{j},$$

$X_{i}$ represents the inputs, where $W_{ij}$ and $b_{j}$ represent connection weights and biased vectors, respectively. Here, a log–sigmoid function is used as an activation function in the feed-forward neural network model, which is given below.

(3) $$f_{j}\left(x\right)=\frac{1}{1+e^{-H_{j}}}.$$

**Figure 1:**
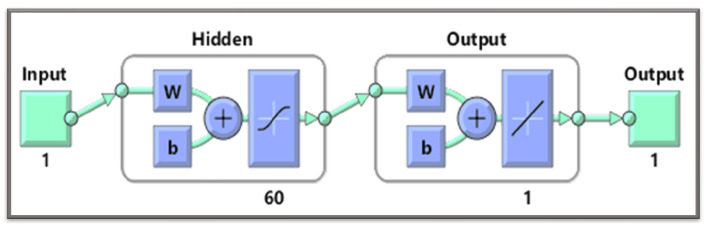
Architecture of an artificial neural network controller.

The MLP, also known as the feed-forward neural network (FNN), is a type of neural network with a hidden layer between the input and output layers. This layer is called the “hidden layer”. The number below the hidden layer represents the number of neurons used inside the network. Figure 1 shows an artificial neural network controller.

A backpropagated Levenberg—Marquardt method is used to train the feed-forward neural network. Local minima can be found using the LM algorithm, which is built-in in many applications.

**Figure 2:**
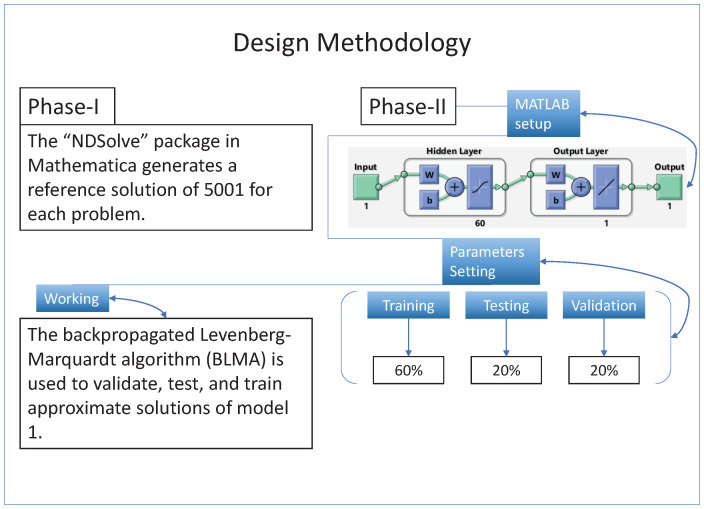
Working mechanism of the NN-BLMA for solving the nonlinear model of vector-borne viral plant disease dynamics.

Additionally, NN-BLMA is implemented in two phases. Figure 2 depicts the Algorithm’s whole workflow, including all of its steps.

- For collecting the initial reference data set, we solve the model (1) numerically by using a state-of-the-art technique. Here we use the RK−4 method, which commonly gave better results, in Mathematica using the “NDSolve” package. The numerical technique generates 5001 in the range of [0,50] with a 0.01 stepsize.
- After that, the NN-BLMA is executed by using “nftool”, a built-in MATLAB tool, to train, validate and test the targets (reference data set). The design technique uses 60% of the targets for training and 20% each for validation and testing. The maximum iteration is set to 1000 with a 60 number of neurons. Table 2 presents the parameters for the design scheme execution, and Algorithm 1 is the pseudo-code of the designed NN-BLMA.

The novel machine learning of NN-BLMA is easy to apply, handles nonlinear problems, and is also a gradient-free technique that converges faster than other machine learning technique [45,46,47,48].
**Algorithm 1:** Pseudocode of NN-BLMA.**Starting of NN-BLMA****Construction**: Construct inputs and reference data set using RK-4 method in Mathematica**Data selection**: Input and target data must be selected in non-linear format, i.e., matrices.**Startup**: Taking number of neurons and distributing the reference data set into training, testing and validation60 Hidden neurons60% data for training20% data for testing20% data for validation**Architecture**: Each input is given a weight, and the input to the transfer function is formed by adding the weights of all of the inputs together along with the bias.**Stopping criteria**: If all of the conditions listed below are met, the previous process will end automatically.Mu reach to its maximum valueNumber of iteration reaches to maximumPerformance value reaches to minimumValidation’s performance became less then maximum failGradient’s performance dropped below minimum gradientThe network is generalised using training data. If the outputs are good, proceed to Saving Output; otherwise, retrain the network.**Retraining**: Change the startup parameters and train the network again**Saving outputs**: End the process by saving the results graphically as well as numerically**Ending of NN-BLMA**

## 4. Numerical Experimentation and Discussion

**Figure 3:**
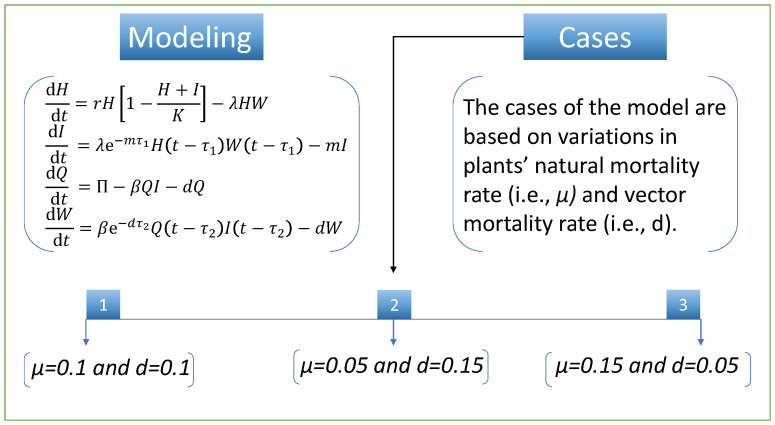
Vector-borne viral plant disease dynamics’ model with its different cases.

To study the design algorithms’ performance and efficiency, we discuss various cases of the nonlinear model of vector-borne viral plant disease dynamics. The cases are based on variation in two parameters (i.e., plants’ natural mortality rate, μ, and vector mortality rate, *d*). We set the same numerical value for both parameters in the first case. In case two, there is a slight decrease in the μ parameter and a slight increase in the parameter *d*, while in the third case, there is an increase in the parameter μ and a decrease in the *d* parameter compared with the first case. Figure 3 illustrates the mathematical model and the cases detail for vector-borne viral plant disease dynamics.

**Figure 4:**
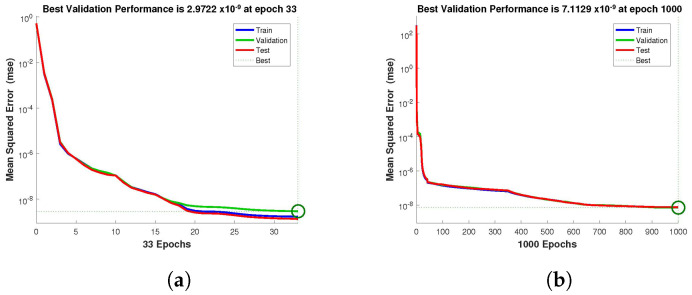

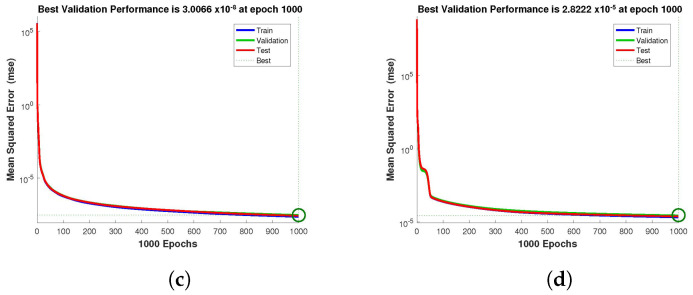
NN-BLMA MSE for healthy and infected plants, and infected and uninfected whitefly of case 1. (**a**) *H*(*t*). (**b**) *I*(*t*). (**c**) *Q*(*t*). (**d**) *W*(*t*).

**Figure 5:**
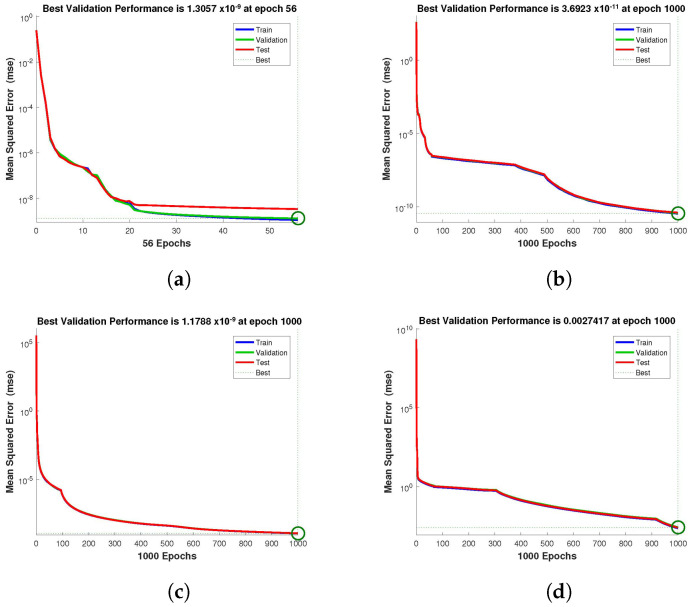
NN-BLMA MSE for healthy and infected plants, and infected and uninfected whitefly of case 2. (**a**) *H*(*t*). (**b**) *I*(*t*). (**c**) *Q*(*t*). (**d**) *W*(*t*).

**Figure 6:**
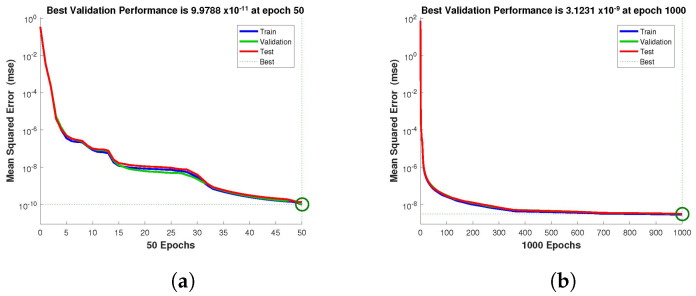

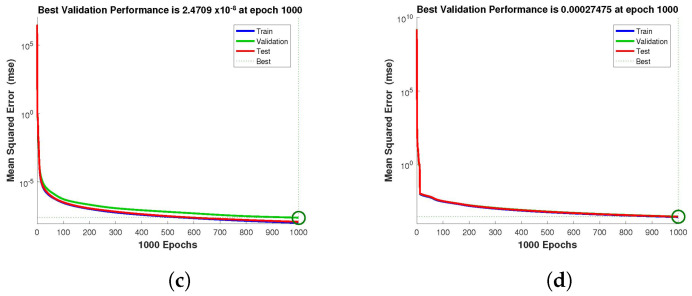
NN-BLMA MSE for healthy and infected plants, and infected and uninfected whitefly of case 3. (**a**) *H*(*t*). (**b**) *I*(*t*). (**c**) *Q*(*t*). (**d**) *W*(*t*).

The design technique generates output data sets with probabilities of 60% of the sample data for testing, 20% for training, and 20% for validation. The performance graph of the design technique shows us its mean squared error (MSE). Figure 4, Figure 5 and Figure 6 depict the best validation performance provided by the design technique because the error is minimized after some epochs of training but may increase on the validation data set as the network begins to overfit the training data. The training is halted after six consecutive rises in the validation error, and the best performance is picked from the epoch with the lowest validation error. The case 1 performance values are in the range of $2.9721\times 10^{-9}$, $7.1129\times 10^{-9}$, $3.0066\times 10^{-8}$ and $2.8222\times 10^{-5}$. Similarly, the case 2 and case 3 performance values are in the range of $1.3057\times 10^{-9}$, $3.6923\times 10^{-11}$, $1.17878\times 10^{-9}$, $1.9703\times 10^{-4}$, and $9.9788\times 10^{-11}$, $3.1230\times 10^{-9}$, $2.4709\times 10^{-8}$, $2.7474\times 10^{-4}$, respectively.

**Figure 7:**
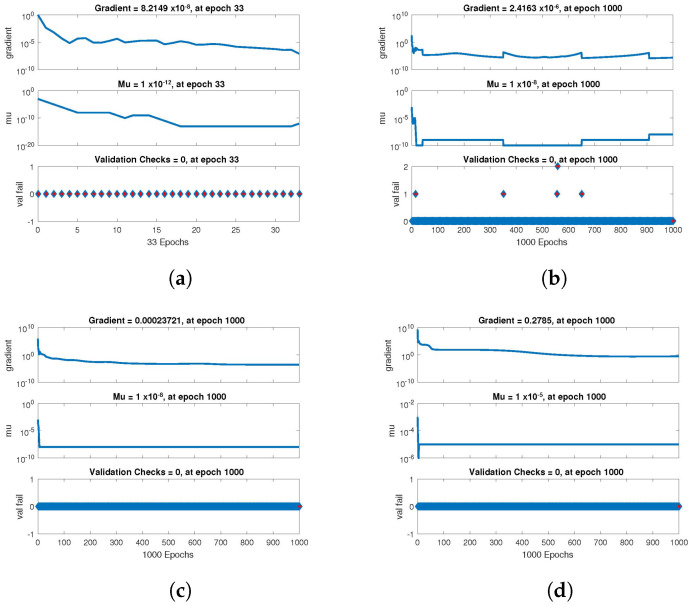
Value of gradient, mu and validation check of NN-BLMA for case 1. (**a**) *H*(*t*). (**b**) *I*(*t*). (**c**) *Q*(*t*). (**d**) *W*(*t*).

**Figure 8:**
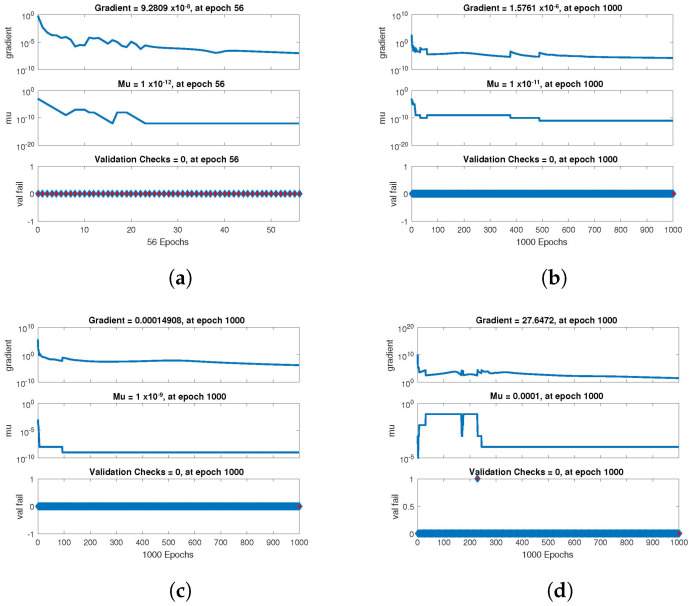
Value of gradient, mu and validation check of NN-BLMA for case 2. (**a**) *H*(*t*). (**b**) *I*(*t*). (**c**) *Q*(*t*). (**d**) *W*(*t*).

**Figure 9:**
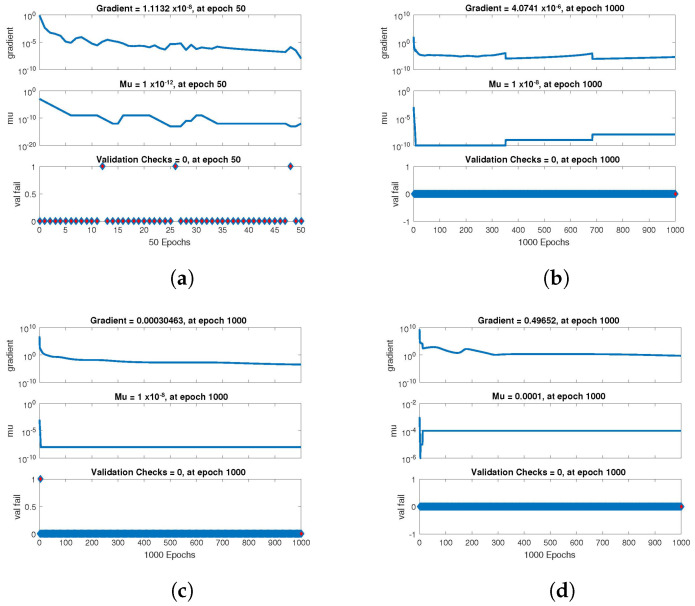
Value of gradient, mu and validation check of NN-BLMA for case 3. (**a**) *H*(*t*). (**b**) *I*(*t*). (**c**) *Q*(*t*). (**d**) *W*(*t*).

**Figure 10:**
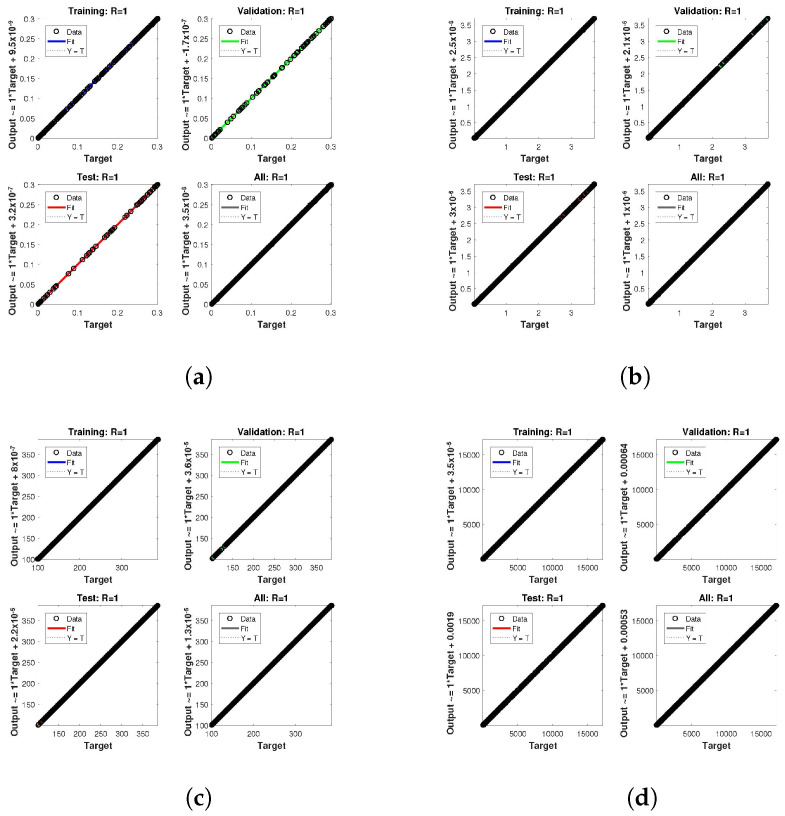
Analysis of regression of the design NN-BLM algorithm for case 1. (**a**) *H*(*t*). (**b**) *I*(*t*). (**c**) *Q*(*t*). (**d**) *W*(*t*).

**Figure 11:**
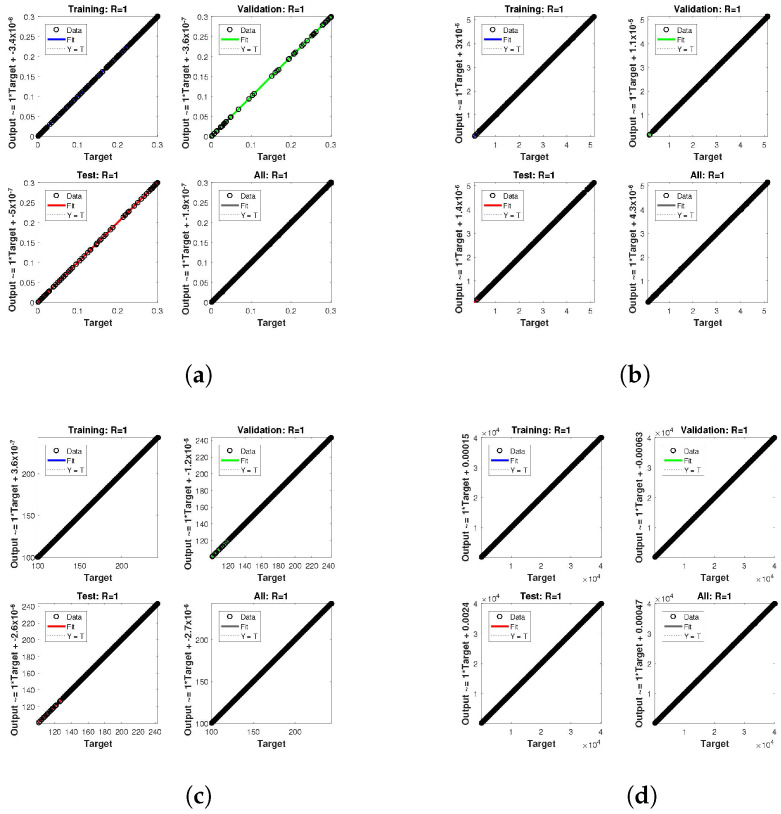
Analysis of regression of the design NN-BLM algorithm for case 2. (**a**) *H*(*t*). (**b**) *I*(*t*). (**c**) *Q*(*t*). (**d**) *W*(*t*).

**Figure 12:**
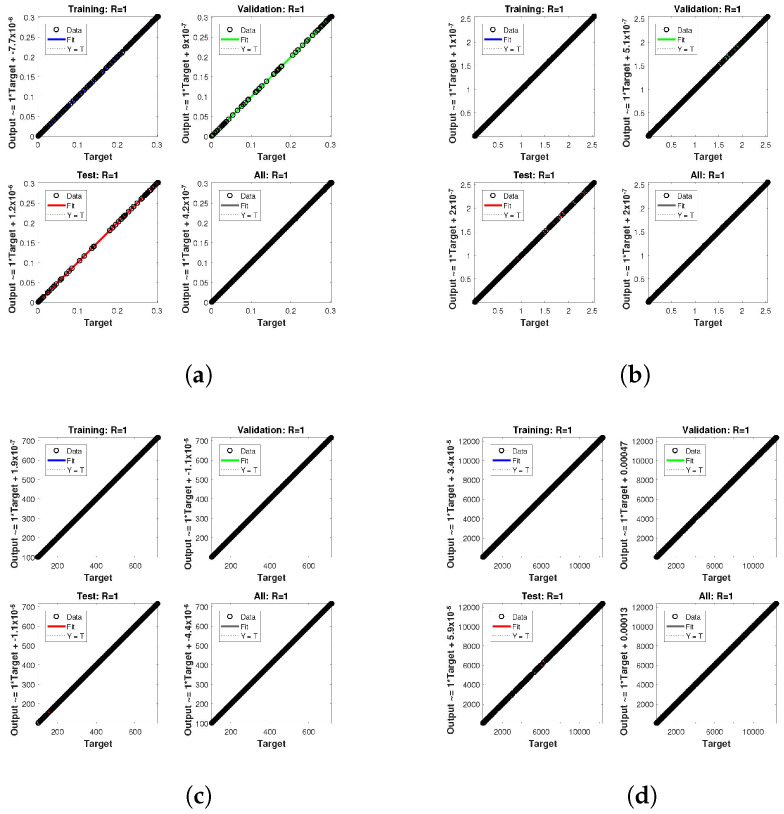
Analysis of regression of the design NN-BLM algorithm for case 3. (**a**) *H*(*t*). (**b**) *I*(*t*). (**c**) *Q*(*t*). (**d**) *W*(*t*).

The statistical performance of all the cases in gradient, mu, and validation failures are illustrated in Figure 7, Figure 8 and Figure 9. The gradient values for the case 1 lie in between $8.2149\times 10^{-8}$, $2.4163\times 10^{-6}$, $2.3721\times 10^{-4}$ and 0.2785, whereas the values for case 2 and case 3 are $9.2809\times 10^{-8}$, $1.5761\times 10^{-6}$, $1.4908\times 10^{-4}$, 27.6472, and $1.1132\times 10^{-8}$, $4.0741\times 10^{-6}$, $3.0463\times 10^{-4}$, 0.49652, respectively. The mu values for all the cases lie in the range $10^{-4}$ to $10^{-13}$. The network output concerning the target for the training, validation, and test sets is shown on the regression plot. The data must fall on a 45-degree line where the network outputs and targets are equal for a perfect match. When the data fall on a 45 degree, the regression plot gives us a value of R=1. This article shows the regression analysis of all the cases in Figure 10, Figure 11 and Figure 12. From the figures, regression values are 1 for all cases, which perfectly matches the network and the targets.

The tables below provide the data information provided by the computing system. The tables show the best performance values in training, testing, validation, etc. Table 3 displays the best performance data for case 1, while the best performance data for case 2 and case 3 are displayed in Table 4 and Table 5, respectively. These tables also show the hidden neuron count, iterations, and time spent.

**Figure 13:**
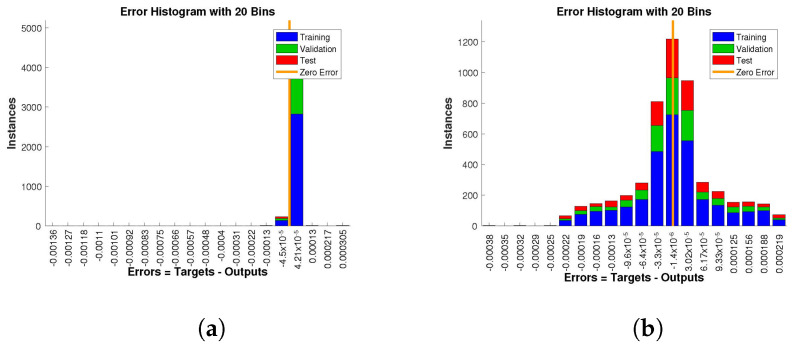

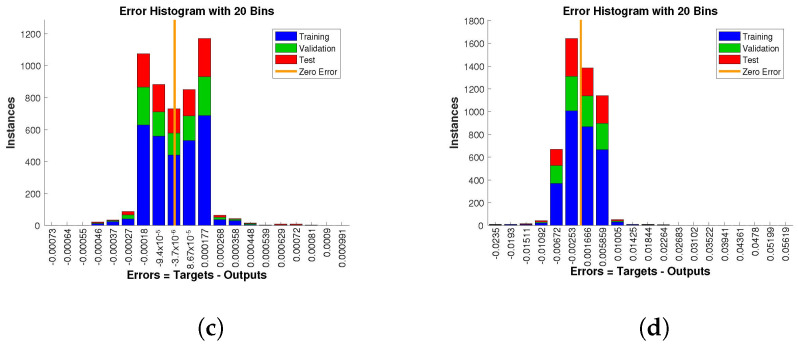
Analysis of the error histogram in terms of the target data and the approximate solutions for case 1. (**a**) *H*(*t*). (**b**) *I*(*t*). (**c**) *Q*(*t*). (**d**) *W*(*t*).

**Figure 14:**
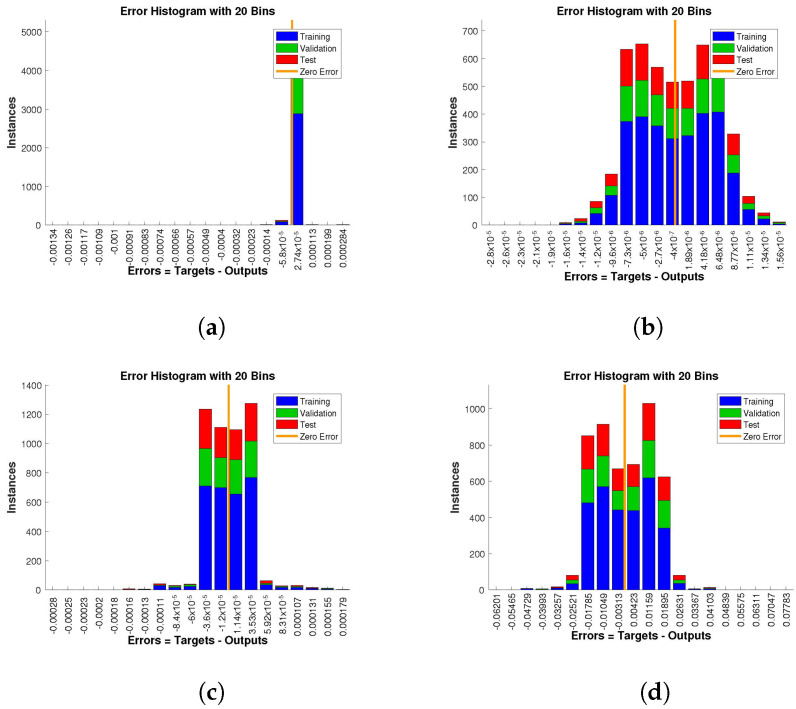
Analysis of the error histogram in terms of the target data and the approximate solutions for case 2. (**a**) *H*(*t*). (**b**) *I*(*t*). (**c**) *Q*(*t*). (**d**) *W*(*t*).

**Figure 15:**
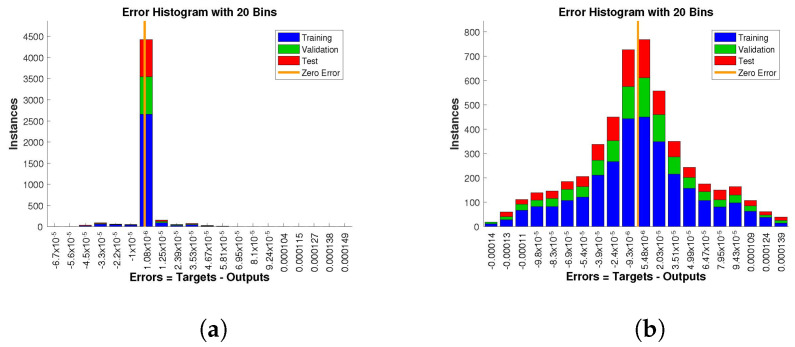

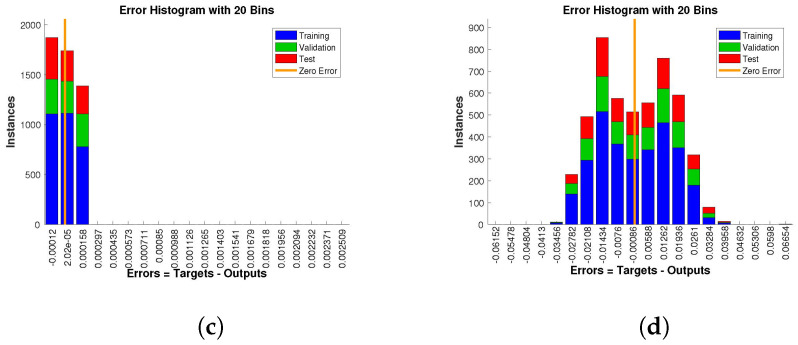
Analysis of the error histogram in terms of the target data and the approximate solutions for case 3. (**a**) *H*(*t*). (**b**) *I*(*t*). (**c**) *Q*(*t*). (**d**) *W*(*t*).

The histogram of errors between targets and outputs after training a neural network is shown in Figure 13, Figure 14 and Figure 15. Different color bars show the errors in the training, validation, and testing data. The error bars in which most of the points lie are very close to the zero error line, which means targets and the outputs are well matched and have the fewest errors, which shows the accuracy of our design technique. The error values for case 1 lie in the range $10^{-3}$ to $10^{-4}$, $10^{-4}$ to $10^{-6}$, $10^{-4}$ to $10^{-6}$, and $10^{-2}$ to $10^{-3}$. For case 2 and case 3 the error values lie in the range $10^{-4}$ to $10^{-5}$, $10^{-5}$ to $10^{-7}$, $10^{-4}$ to $10^{-5}$, $10^{-3}$ to $10^{-4}$, $10^{-2}$ to $10^{-3}$, $10^{-4}$ to $10^{-6}$, $10^{-4}$ to $10^{-6}$, $10^{-3}$ to $10^{-5}$, and $10^{-2}$ to $10^{-3}$, respectively.

**Figure 16:**
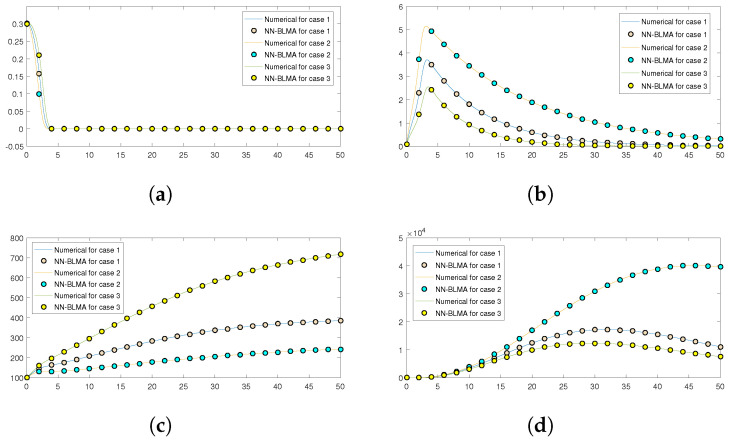
Numerical solutions’ comparison of NN-BLMA with the solution obtained with other numerical methods. (**a**) Healthy plants *H*(*t*). (**b**) Infected plants *I*(*t*). (**c**) Uninfected whiteflies *Q*(*t*). (**d**) Infected whiteflies *W*(*t*).

Further, Figure 16 compares the numerical solution of the model obtained by the “NDSolve” package in Mathematica (targets) to the solution obtained by executing NN-BLMA (outputs). The solid lines show the solution obtained by solving the model numerically by the “NDSolve” package in Mathematica, while the circles show the solution by NN-BLMA. In the figure, we see that the solutions obtained from NN-BLMA come exactly on the targets’ solutions lines, which shows how accurate our design technique is. These figures also indicate the model’s variation due to some parameters in the model. It is obvious from the figures that healthy plants and uninfected whiteflies rise when there is an increase in plant mortality rate and a drop in vector mortality rate. In contrast, a drop in plant mortality rate and an increase in vector mortality rate leads to a rise in infected plants and whiteflies. The comparison of statistical data given by the ’NDSlove’ package in Mathematica with the outputs of NN-BLMA is illustrated in the tables below. Table 6 illustrates the comparative analysis of both the solutions for case 1, while the comparison for case 2 and case 3 are illustrated in Table 7 and Table 8, respectively.

## 5. Conclusions

In this paper, we analyzed a mathematical model for persistent vector-borne viral plant disease dynamics. The model includes equations for healthy and infected plants and uninfected and infected whiteflies. The selected set of parameters for numerical simulation is for the cause of the mosaic disease in cassava. To see the impact of variation in the mortality parameters on the model, we made different cases in which we vary both plant and vector mortality parameters. The reference data (targets) for NN-BLMA were generated by solving the model numerically for all the cases in Mathematica. The designed technique uses the targets to train, test, and validate the ANN and to see the impact of variation in plants’ natural and vectors’ mortality rates. The key points concluded from the study are given below.

- From the study, we see an increase in the mortality rate of plants, along with a decrease in the mortality rate of vectors, increases in healthy plants and uninfected whiteflies, and decreases in infected plants and infected whiteflies. In contrast, a drop in the mortality rate of plants and an increase in the mortality rate of vectors results in a decrease in healthy plants and uninfected whiteflies and an increase in the number of infected plants and infected whiteflies.
- Further, the accuracy of the design technique is illustrated through extensive graphical and tabular data, which include the best performance in terms of the mean squared error, histogram, and regression analyses.

## Figures and Tables

**Table 2:** The NN-BLMA parameters settings for implementation.

**Index**	Learning Methodology	Training	Validation	Testing	Hidden Neuron	Max. Iteration
**Description**	Levenberg—Marquardt	60%	20%	20%	60	1000

**Table 3:** Performance values of the design NN-BLMA, and time spent by the computing system to obtain solutions for case 1.

	*H*(*t*)	*I*(*t*)	*Q*(*t*)	*W*(*t*)
Training	$1.68\times 10^{-9}$	$7.50\times 10^{-9}$	$2.29\times 10^{-8}$	$2.10\times 10^{-5}$
Validation	$2.97\times 10^{-9}$	$7.11\times 10^{-9}$	$3.01\times 10^{-8}$	$2.82\times 10^{-5}$
Testing	$1.35\times 10^{-9}$	$7.61\times 10^{-9}$	$2.67\times 10^{-8}$	$2.43\times 10^{-5}$
Gradient	$8.2149\times 10^{-8}$	$2.4163\times 10^{-6}$	$2.3721\times 10^{-4}$	0.2785
Mu	$1\times 10^{-12}$	$1\times 10^{-8}$	$1\times 10^{-8}$	$1\times 10^{-5}$
Epoches	33	1000	1000	1000
Regression	1	1	1	1
Time	30	30	30	30

**Table 4:** Performance values of the design NN-BLMA, and time spent by the computing system to obtain solutions for case 2.

	*H*(*t*)	*I*(*t*)	*Q*(*t*)	*W*(*t*)
Training	$1.12\times 10^{-9}$	$3.41\times 10^{-11}$	$1.15\times 10^{-9}$	$1.79\times 10^{-4}$
Validation	$1.31\times 10^{-9}$	$3.69\times 10^{-11}$	$1.18\times 10^{-9}$	$1.97\times 10^{-4}$
Testing	$3.38\times 10^{-9}$	$4.01\times 10^{-11}$	$1.18\times 10^{-9}$	$2.01\times 10^{-4}$
Gradient	$9.2809\times 10^{-8}$	$1.5761\times 10^{-6}$	$1.4908\times 10^{-4}$	27.6472
Mu	$1\times 10^{-12}$	$1\times 10^{-11}$	$1\times 10^{-9}$	$1\times 10^{-4}$
Epoches	56	1000	1000	1000
Regression	1	1	1	1
Time	30	30	30	30

**Table 5:** Performance values of the design NN-BLMA, and time spent by the computing system to obtain solutions for case 3.

	*H*(*t*)	*I*(*t*)	*Q*(*t*)	*W*(*t*)
Training	$9.84\times 10^{-11}$	$2.84\times 10^{-9}$	$1.04\times 10^{-8}$	$2.53\times 10^{-4}$
Validation	$9.98\times 10^{-11}$	$3.12\times 10^{-9}$	$2.47\times 10^{-8}$	$2.75\times 10^{-4}$
Testing	$1.38\times 10^{-10}$	$3.16\times 10^{-9}$	$1.23\times 10^{-8}$	$2.69\times 10^{-4}$
Gradient	$1.1132\times 10^{-8}$	$4.0741\times 10^{-6}$	$3.0463\times 10^{-4}$	0.49652
Mu	$1\times 10^{-12}$	$1\times 10^{-8}$	$1\times 10^{-8}$	$1\times 10^{-4}$
Epochs	50	1000	1000	1000
Regression	1	1	1	1
Time	30	30	30	30

**Table 6:** Comparative analysis of numerical solution with the solutions obtained from NN-BLMA for case 1.

	*H*(*t*)		*I*(*t*)		*Q*(*t*)		*W*(*t*)	
***t***	**Numerical**	**NN-BLMA**	**Numerical**	**NN-BLMA**	**Numerical**	**NN-BLMA**	**Numerical**	**NN-BLMA**
0	0.3	0.301402499	0.1	0.100121	100	100.0008	5	4.941716
0.5	0.295659	0.295796496	0.659197	0.659184	114.0068	114.0061	4.828814	4.811659
1	0.270171	0.270148397	1.121609	1.121648	126.3683	126.3679	5.584575	5.598602
1.5	0.225534	0.225481705	1.610816	1.610781	137.0851	137.0855	9.607768	9.620728
2	0.158531	0.158487646	2.28979	2.289811	145.892	145.892	21.79219	21.80038
2.5	0.078258	0.078217137	3.113732	3.113741	152.3632	152.3629	51.27621	51.28554
3	0.020596	0.020563851	3.647005	3.646977	156.6824	156.6827	108.024	108.0293
3.5	0.002145	0.002150992	3.673769	3.673765	159.9924	159.9922	193.49	193.4844
4	$6.66\times 10^{-5}$	$6.67153\times 10^{-5}$	3.500676	3.646977	163.2073	163.2072	303.6808	303.6772

**Table 7:** Comparative analysis of numerical solution with the solutions obtained from NN-BLMA for case 2.

	*H*(*t*)		*I*(*t*)		*Q*(*t*)		*W*(*t*)	
***t***	**Numerical**	**NN-BLMA**	**Numerical**	**NN-BLMA**	**Numerical**	**NN-BLMA**	**Numerical**	**NN-BLMA**
0	0.3	0.301386	0.1	0.100008961	100	100.0002874	5	4.91848547
0.5	0.2877	0.287758	1.021413	1.021411462	111.136459	111.1363243	4.74700621	4.704905202
1	0.243996	0.243941	1.778091	1.778087194	120.0058588	120.0057731	5.886795798	5.906826879
1.5	0.178663	0.178615	2.615206	2.615204348	126.6983972	126.6984875	11.85176957	11.86964906
2	0.099554	0.099532	3.721032	3.721035712	130.919435	130.9195312	29.55952416	29.56956409
2.5	0.033451	0.033442	4.748642	4.748643211	132.5695704	132.5695372	69.40014059	69.40036895
3	0.005173	0.005141	5.142219	5.142218201	132.6625165	132.6624534	137.4934921	137.5035161
3.5	0.000284	0.000276	5.084467	5.084461504	132.5157456	132.515754	231.7495915	231.7676383
4	$4.2\times 10^{-6}$	$1.5\times 10^{-5}$	4.939332	4.939329075	132.6092218	132.6092389	350.3411185	350.3583379

**Table 8:** Comparative analysis of numerical solution with the solutions obtained from NN-BLMA for case 3.

	*H*(*t*)		*I*(*t*)		*Q*(*t*)		*W*(*t*)	
***t***	**Numerical**	**NN-BLMA**	**Numerical**	**NN-BLMA**	**Numerical**	**NN-BLMA**	**Numerical**	**NN-BLMA**
0	0.3	0.29984487	0.1	0.10015	100	100.0001	5	4.930092
0.5	0.300552	0.30057977	0.43618653	0.43611909	116.834648	116.8346	4.92687225	4.91208
1	0.287377	0.28740484	0.71468896	0.71462628	132.625296	132.6252	5.47558404	5.499553
1.5	0.259799	0.25980546	0.99348118	0.99348179	147.331811	147.3318	8.22851282	8.216368
2	0.21123	0.21120628	1.36866609	1.3687244	160.767638	160.7677	16.453172	16.44464
2.5	0.136805	0.13676428	1.88979607	1.88976049	172.537448	172.5374	36.7607881	36.7496
3	0.056486	0.05643249	2.38574334	2.38574809	182.351472	182.3515	79.3186069	79.29844
3.5	0.010986	0.01094595	2.54620998	2.54620843	190.689067	190.6891	150.762051	150.7577
4	0.000742	0.00072402	2.42175547	2.42176342	198.537627	198.5377	248.404668	248.4245

## Data Availability

The data supporting this study’s findings are available upon reasonable request from the corresponding author. The MATLAB code and a data file generated by the NDSolve Mathematica packager are available at author’s GitHub account: https://github.com/sulaiman513/AWKUM-Optimization-Lab, accessed on 1 September 2022.

## Nomenclature

ANN	Artificial neural network
NN-BLMA	Backpropagated neural network based Levenberg—Marquardt Algorithm
MLP	Multilayer perceptron
FNN	Feed-forward neural network
BMV	Beet mosaic virus
ACMV	African cassava mosaic virus
TMV	Tobacco mosaic virus
BGMV	Bean golden mosaic virus
DDE	Delay differential equations
MSE	Mean squared error
*H*	Healthy plants
*I*	Infected plants
*Q*	Uninfected whiteflies
*W*	Infected whiteflies
*r*	Net growth rate of plants
*k*	Carrying capacity
λ	Rate of disease transmission from infected vector to healthy plant
μ	Plants natural mortality rate
$m_{1}$	Mortality of infected plants
Π	Cumulative birth or immigration rate of vector population
β	Transmission rate between diseased vector and healthy plant
*d*	Vector mortality rate
